# Supplementary material for: Rapid turnover of life-cycle-related genes in the brown algae
Source: Genome Biol. 2019 Feb 14;20:35. doi: 10.1186/s13059-019-1630-6 (PMC6374913; doi:10.1186/s13059-019-1630-6)
Supplement: Supplementary file 3 — Protein alignments, corresponding Gblocks html file and CDS sequences for the lineage conserved gametophyte-biased genes. (ZIP 2109 kb) [file 13059_2019_1630_MOESM3_ESM.zip › Con_GA_alignments/Ec-12_004770.aa_ali.fasta-gb.htm]

Ec-12\_004770.aa.fasta.trans.aa\_ali.fasta


## Gblocks 0.91b Results

Processed file: **Ec-12\_004770.aa.fasta.trans.aa\_ali.fasta**  
Number of sequences: **2**  
Alignment assumed to be: **Protein**  
New number of positions: **420** (selected positions are underlined in blue)

```
                         10        20        30        40        50        60
                 =========+=========+=========+=========+=========+=========+
Ec-12_004770     MGTSAEHGGGRITVCCRIRPLGTS---TERVPIGTNHRCLWVHSAEPSSVLAGQSGS---
MSTRG.38150.1    --------XRKVKVCCRIRPLNTTSTATETNVTDVGDSRLHIKSAGPSLALVSRSGSRSR
                              ##########                ##################   


                         70        80        90       100       110       120
                 =========+=========+=========+=========+=========+=========+
Ec-12_004770     ---SSSSTARWGARTVRGGARN-----------DRWGFTFDDVLEAGCSQEEVYRKCAKD
MSTRG.38150.1    ECSSSSKSSSPGSKKAGQGTSGDSETEGLRRKRGRWEFTFDDVLEDGCSQEEVYRKCATD
                    ################               ##########################


                        130       140       150       160       170       180
                 =========+=========+=========+=========+=========+=========+
Ec-12_004770     ILESALNGVNGTIMAYGCTGAGKSYTMMGS-DRDGVDDANRSGICARALRDVFREAARRR
MSTRG.38150.1    IVESVLKGINGTIMAYGCTGAGKSYTMMGAAERDGIDETRQSGICARALRNIFQEANRRR
                 #############################   ############################


                        190       200       210       220       230       240
                 =========+=========+=========+=========+=========+=========+
Ec-12_004770     RGNDKPAITIQMSFVEIYNEQLFDLLLPPEAYSSAN--QQQRRFSVYGGK--ESAVGGKR
MSTRG.38150.1    RRTG-STYTVQMSFVEIYNEQLFDLLLSPEASSAKNKHHHQYRFNVYGGNGNGSAIGGKR
                 #       ############################                 #######


                        250       260       270       280       290       300
                 =========+=========+=========+=========+=========+=========+
Ec-12_004770     NTPLSSIQRLVGELEVGSAGGSGSGLHAPLTTTPGARQQMADLAIYERPDGSTYVKGLRV
MSTRG.38150.1    KMPASLAQRLETDRAV------G----------SGCDGEVLDLAIYERTDGSTYVKGLSL
                 ################                  ##########################


                        310       320       330       340       350       360
                 =========+=========+=========+=========+=========+=========+
Ec-12_004770     HTVSTEGEALRYLRCGQVAREVAEHQFNRVSSRSHAVLTVHLAMSGGDEGKTTDEQNAGR
MSTRG.38150.1    LLVGNEEEALRILRAGQRAREVAEHQFNHASSRSHAVVTVHLTTHGGSE----DDIKCGK
                 #################################################           


                        370       380       390       400       410       420
                 =========+=========+=========+=========+=========+=========+
Ec-12_004770     GVAKGPGRWIGGGGGGGGGGGGVATTSKLNLVDLAGSEKSATG--ATGANAVLHREGRFI
MSTRG.38150.1    --------DEGGRGGGAGGGGGDVTASKLNLVDLAGSEKSVIEGATDGDAAILHREGRMI
                           ##############################       #############


                        430       440       450       460       470       480
                 =========+=========+=========+=========+=========+=========+
Ec-12_004770     NKSLTFLEQVTMALADQTREHVPFRSSKLTHFLKDSLGGNCQTLMVACIWPRPDLLQQSL
MSTRG.38150.1    NKSLTFLEQVIIALADHKREHVPFRSSKLTHFLK------------------------SL
                 ##################################                        ##


                        490       500       510       520       530       540
                 =========+=========+=========+=========+=========+=========+
Ec-12_004770     ATLKFAARMRCVKNSPIVNERSWSADDEIARLRSQVDLLETQLAAQQAAGGGGRAGCESP
MSTRG.38150.1    ATLKFAARMRCVKTTPLIGTRIWSAEDEITSLRRQVSQLQARLALSVDRRGVSTASTSSA
                 ############################################################


                        550       560       570       580       590       600
                 =========+=========+=========+=========+=========+=========+
Ec-12_004770     AYADNVPQHDRRRRRRVWEEAGRFVLEGRSNNNCGGRRRATAEATGGRRRRGKRSSGLQA
MSTRG.38150.1    AGST-----------------------------------ATDVAAGGRRR----------
                 #                                      ###########          


                        610       620       630       640       650       660
                 =========+=========+=========+=========+=========+=========+
Ec-12_004770     TAACGSREDGDGRKSDSLTTVPSEEGDDDEEEEEEEEEEEMAEGEAGDDAGHRGRSNKMA
MSTRG.38150.1    ------------------------------------------------------------
                                                                             


                        670       680       690       700       710       720
                 =========+=========+=========+=========+=========+=========+
Ec-12_004770     EAKTGGGKLAVAAAVRSAVDAVVGRSNRDIDSPESSRGGSRQEDGRREGTRRYEGDRLPF
MSTRG.38150.1    ----------------------------ALPST---------------------------
                                                                             


                        730       740       750       760       770       780
                 =========+=========+=========+=========+=========+=========+
Ec-12_004770     IGSRSDPSTDTWIVARAPGGASCSEEFGGDGDGDGPDGSRPSERLGGGICTTLSNKSGGA
MSTRG.38150.1    ------------------------------------------------------------
                                                                             


                        790       800       810       820       830       840
                 =========+=========+=========+=========+=========+=========+
Ec-12_004770     GGGSAAKKADNKEGGASRRRSVPGYGGDEDVSNAESTAVTEDDWTGVTESHGTGGEIEET
MSTRG.38150.1    ----------------ARRR---------------------------------GGIMEEA
                                                                      #######


                        850       860       870       880       890       900
                 =========+=========+=========+=========+=========+=========+
Ec-12_004770     SRRRRRRRCRRRHHLGGAFELFKISEGKQSNSRLQDAKAAVQRVRSLASDLTGRVNDAKG
MSTRG.38150.1    GRFVLQRRTAVAKSVSAA--------GQRGSSLLRE------------------------
                 ########                                                    


                        910       920       930       940       950       960
                 =========+=========+=========+=========+=========+=========+
Ec-12_004770     GIDVARAQLRQHQQRKGSQGDGDECEEEKEREEHISSATLALTAQLKSRKREYRVLFEKL
MSTRG.38150.1    ------------------------------------------------------------
                                                                             


                        970       980       990      1000      1010      1020
                 =========+=========+=========+=========+=========+=========+
Ec-12_004770     AGAKAELRKLRQDKQKELAQLAADFECQQEKSFRSRSSSNSNNRSTDTSASFLPALRPTA
MSTRG.38150.1    ------------------------------------------------------------
                                                                             


                       1030      1040      1050      1060      1070      1080
                 =========+=========+=========+=========+=========+=========+
Ec-12_004770     AMSLAGGGCAAATTTARCGEENRGDGGGGGGSMSGDGAGDVGGRHLVASSSVASPSAARA
MSTRG.38150.1    ----------------------RGESGGEEG-----GG--------------------KD
                                                                             


                       1090
                 =========+===
Ec-12_004770     KAVVLQQPRAASF
MSTRG.38150.1    IGSLLTQPEE---
```

```
Parameters used
Minimum Number Of Sequences For A Conserved Position: 2
Minimum Number Of Sequences For A Flanking Position: 2
Maximum Number Of Contiguous Nonconserved Positions: 8
Minimum Length Of A Block: 10
Allowed Gap Positions: None
Use Similarity Matrices: Yes
```

```
Flank positions of the 13 selected block(s)
Flanks: [14  23]  [40  57]  [64  79]  [95  149]  [153  181]  [189  216]  [234  256]  [275  349]  [371  400]  [408  454]  [479  541]  [580  590]  [834  848]  

New number of positions in Ec-12_004770.aa.fasta.trans.aa_ali.fasta-gb:  420  (38% of the original 1093 positions)
```
